# Low-Temperature Transcriptional Responses in Selected pESI-Positive *Salmonella* Infantis Isolates with Contrasting Antimicrobial Resistance Phenotypes: An Exploratory Study

**DOI:** 10.3390/foods15193512

**Published:** 2026-10-01

**Authors:** Arife Ezgi Telli, Yusuf Biçer, Gamze Turkal, Ahmet Gökhan Coşkun, Nihat Telli

**Affiliations:** 1 Department of Food Hygiene and Technology, Faculty of Veterinary Medicine, Selcuk University, Konya 42130, Türkiye; yusufbicer@selcuk.edu.tr (Y.B.); gamze.kabak@selcuk.edu.tr (G.T.); 2 Food and Feed Branch Directorate, Balıkesir Provincial Directorate of Agriculture and Forestry, Balıkesir 10020, Türkiye; ahmetgokhancoskun@hotmail.com; 3 Department of Food Processing, Vocational School of Technical Sciences, Konya Technical University, Konya 42130, Türkiye; ntelli@ktun.edu.tr

**Keywords:** *Salmonella* Infantis, pESI plasmid, cold stress, gene expression

## Abstract

Refrigeration and freezing are commonly used preservation methods in the control of foodborne pathogens. However, the transcriptional responses of *Salmonella* Infantis to low-temperature conditions remain incompletely understood. In this study, the effect of low-temperature stress on the expression of pESI-related *repA*, *ipf*, and K88-like genes, as well as *cspA*, involved in cold shock response, and *ompF*, associated with environmental stress adaptation, was investigated in three pESI-positive, antibiotic-susceptible and three pESI-positive multi-antibiotic-resistant (MDR) *S.* Infantis isolates. Isolates were exposed to 4 °C and −20 °C conditions for 24 h, while isolates incubated at 37 °C were considered as the control group. Relative expression levels of genes were determined using the SYBR Green-based RT-qPCR method. Mean transcriptional patterns differed between the two selected isolate groups, most prominently at 4 °C. Relative to their respective 37 °C controls, all five target genes showed decreased mean expression in susceptible isolates but increased mean expression in MDR isolates at 4 °C. At −20 °C, group-mean expression was increased across all five targets in the MDR group, whereas the susceptible group showed decreased mean expression of the pESI-associated targets *repA*, *ipfA*, and *klf*, but modest increases in *ompF* and *cspA*. A significant temperature-by-phenotype interaction was detected for *ipfA* (*p* = 0.029). Between-group comparisons identified significant differences for the K88-like fimbrial locus (*klf*) and *ompF* at 4 °C. However, larger isolate groups and functional validation studies are needed to elucidate the biological significance of the identified expression changes.

## 1. Introduction

*Salmonella enterica* serovar Infantis (*S.* Infantis) has emerged as one of the most important non-typhoidal *Salmonella* serovars in both human infections and the food chain in recent years. It is recognized as an important cause of foodborne gastroenteritis. Not only its prevalence, but also its adaptability to environmental conditions and increasing antimicrobial resistance profile make it noteworthy [1]. Furthermore, the rise in multi-antibiotic-resistant strains complicates the effectiveness of control strategies [1,2].

The emergence of multidrug-resistant *S.* Infantis has been closely associated with the dissemination of large conjugative pESI-like megaplasmids. These plasmids may carry antimicrobial-resistance determinants, virulence-associated factors, and genes contributing to bacterial fitness under adverse environmental conditions [3,4]. This suggests that the effects of environmental stressors on bacterial gene expression may be shaped not only at the chromosomal level but also through plasmid-mediated genetic elements. In addition to antimicrobial resistance, pESI-like plasmids are thought to enhance bacterial fitness by promoting persistence and adaptation under adverse environmental conditions [5]. Therefore, understanding the transcriptional responses of pESI-associated genes under food-related stress conditions may provide valuable insights into the survival strategies of *S.* Infantis throughout the food chain. Among these plasmid-associated genes, *repA* is involved in plasmid maintenance, whereas *ipfA* and K88-like are associated with fimbrial structures that may contribute to bacterial persistence and adaptation.

It has been reported that pESI-like plasmids can carry varying numbers and contents of antibiotic resistance genes in different isolates; in some cases, these plasmids may contain resistance genes only to a limited extent or may not carry them at all. This reveals that pESI positivity alone cannot be directly associated with a specific resistance phenotype. Therefore, although the presence of pESI-like plasmids is generally associated with multi-antibiotic resistance, it is understood that different isolates may exhibit different antibiotic resistance profiles even though they carry the same plasmid; this difference may vary depending on the gene content, mobile element structure, and genetic context of the plasmid [6,7].

Refrigeration and freezing are among the most widely used preservation methods for controlling microbial growth in the food industry. However, these conditions do not completely eliminate *Salmonella*; instead, they impose environmental stress that triggers a wide range of physiological and transcriptional adaptations [5,8]. Cold stress leads to widespread changes in gene expression in *Salmonella*. In particular, cold shock proteins (*cspA*, *cspB*) and oxidative stress response genes (e.g., *oxyR*, *katG*) have been shown to play important roles in maintaining cellular stability and protein synthesis under low-temperature conditions [9]. Nevertheless, this serotype has been reported to persist under refrigerated conditions, particularly within the poultry production chain, suggesting a remarkable capacity for environmental adaptation [10,11]. The response to cold stress is not limited to cold shock proteins; the changes that low temperatures cause in cell membrane structure and envelope homeostasis make outer membrane-related responses an important component of this process [12]. *ompF* encodes one of the key porins that facilitates the passive passage of small hydrophilic molecules across the outer membrane, and its expression is regulated by the EnvZ/OmpR system, which responds to environmental signals, particularly changes in osmolarity. In *Salmonella* Typhimurium, *ompF* has been shown to be part of the *OmpR*-mediated acid and osmotic stress response [13]. Decreased porin expression or porin loss can contribute to the resistance phenotype in Gram-negative bacteria by restricting the entry of antimicrobial agents into the cell [14,15]. Therefore, *ompF* was selected as a chromosomal target in this study to evaluate the potential link between outer membrane-related stress response and resistance phenotype under low-temperature conditions.

It is noteworthy that studies examining gene expression under low-temperature conditions specific to *S.* Infantis are quite limited in the literature. Existing studies have mostly been conducted on other Salmonella serotypes, and the findings obtained appear to be indirectly interpreted for *S.* Infantis. This situation indicates that serotype-level specific gene expression responses are not sufficiently elucidated and there is a significant knowledge gap in this area [16,17]. Recently, transcriptomic studies have begun to investigate the stress responses of *S.* Infantis; however, these studies have primarily focused on acid, oxidative, or osmotic stress [5], whereas the transcriptional behavior of pESI-associated genes under low-temperature stress remains largely unexplored.

The aim of this study was to compare the effects of refrigerated (4 °C) and frozen (−20 °C) conditions on the expression of selected pESI-associated and chromosomal stress-response genes in pESI-positive susceptible and multidrug-resistant *S.* Infantis isolates.

## 2. Materials and Methods

### 2.1. Origin, Characterization, and Selection of Isolates

Six previously isolated and characterized *Salmonella enterica* serovar Infantis isolates were selected for the present study. The isolates originated from distinct retail chicken samples collected in the Eastern Anatolia region of Türkiye in 2024. The sample sources were chicken thigh (R1, R2, and S2), chicken liver (R3 and S1), and chicken wing (S3).

For the original isolation, samples were analyzed for *Salmonella* according to the USDA-FSIS [18]. Presumptive *Salmonella* isolates were further characterized by conventional biochemical tests, Gram staining, catalase and oxidase tests (Oxoid, UK), and latex agglutination (Thermo Fischer Scientific, South Australia). Molecular confirmation was subsequently performed by PCR. Isolates were first confirmed as *Salmonella* spp. using a species-specific PCR assay, followed by multiplex PCR for differentiation of *S.* Infantis, *S.* Enteritidis, and *S.* Typhimurium.

Following identification as *S.* Infantis, the isolates were screened for three pESI-associated targets, *repA*, *ipfA*, and the K88-like fimbrial locus, by multiplex PCR. Comparative genomic analyses have shown these targets to be highly conserved among pESI/pESI-like plasmids, with all three sequences reported in >99% of the plasmids examined. Based on these findings, a multiplex PCR assay targeting *repA*, *ipfA*, and K88-like sequences was subsequently developed and validated for the detection of pESI-associated *S.* Infantis isolates. The combined detection of these three markers has also been used in subsequent studies as an indicator of pESI/pESI-like plasmid carriage. Accordingly, isolates positive for all three targets were operationally classified as pESI-positive for the purposes of the present targeted study [19,20,21].

Antimicrobial susceptibility was determined by disk diffusion and interpreted using EUCAST Clinical Breakpoint Tables v13.1 (2023) for AMC, AM, ATM, and FEP, and CLSI M100-Ed33 (2023), Enterobacterales Table 2A, for CPD and CXM (Supplementary Table S1) [22,23]. To maximize phenotypic contrast while maintaining pESI positivity across the study set, three isolates susceptible to all tested antimicrobials (S1–S3) and three isolates resistant to all tested antimicrobials (R1–R3) were selected. Thus, the six isolates represented deliberately selected phenotypic extremes rather than a population-representative collection. Their individual characteristics are summarized in Table 1, and isolate level antimicrobial susceptibility profiles are provided in Supplementary Table S1.

### 2.2. Selection of Target Genes

Five genes representing selected plasmid-associated and chromosomal functions were included in the targeted expression analysis: three pESI-associated targets (*repA*, *ipfA*, and the K88-like fimbrial locus [*klf*]) and two chromosomal stress-response genes (*ompF* and *cspA*). The *repA* gene is involved in plasmid replication and maintenance, whereas *ipfA* and the K88-like fimbrial locus are associated with pESI-encoded fimbrial structures. These targets were selected to examine specific pESI-associated functions, namely plasmid replication and maintenance and fimbrial loci, rather than to represent the overall transcriptional activity of the pESI plasmid. The chromosomal gene *cspA* was included as a marker of the cold-shock response, while *ompF* was selected to evaluate outer-membrane-associated adaptation under low-temperature stress. This targeted five-gene panel was not intended to represent the complete pESI transcriptome or the entire cold-stress response. The primer sequences used for RT-qPCR analyses are presented in Table 2.

### 2.3. Low-Temperature Stress Conditions

Isolates were grown overnight in tryptic soy broth (TSB) at 37 °C. The cultures were adjusted to a turbidity equivalent to a 0.5 McFarland standard, distributed into 10-mL aliquots, and exposed to 37 °C, 4 °C, or −20 °C for 24 h. The 24 h exposure period was selected to evaluate transcriptional profiles following prolonged low-temperature exposure rather than the immediate cold-shock response. Following exposure, samples stored at −20 °C were thawed on ice, and all samples were immediately processed for RNA extraction.

### 2.4. RNA Extraction and Gene Expression Analysis

#### 2.4.1. RNA Isolation

After 24 h of incubation under experimental conditions (37 °C, 4 °C, and −20 °C), bacterial cells from each biological replicate were harvested by centrifugation at 12,000× *g* for 5 min at 4 °C. Total RNA isolation was performed using PureZOL™ RNA Isolation Reagent (Bio-Rad Laboratories, Hercules, CA, USA) in accordance with the manufacturer’s recommendations.

Briefly, cell pellets were homogenized in 1 mL of PureZOL reagent and kept at room temperature for 5 min to ensure complete dissociation of nucleoprotein complexes. Then, 200 μL of chloroform was added, and samples were mixed vigorously and centrifuged for 15 min at 12,000× *g* at 4 °C for phase separation. The resulting supernatant was transferred to a new RNase-free tube, and RNA was precipitated by the addition of 500 μL isopropanol and incubated at −20 °C. RNA pellets were collected by centrifugation at 12,000× *g* for 10 min, washed with 75% ethanol, briefly air-dried, and dissolved in RNase-free water. The resulting RNA samples were treated with RNase-free DNase I (Thermo Scientific, Waltham, MA, USA) according to the manufacturer’s instructions to remove residual genomic DNA.

RNA quantity and purity were evaluated based on the A260/A280 absorbance ratio using the NanoDrop spectrophotometer. RNA integrity was confirmed by 1% agarose gel electrophoresis. RNA isolation was performed independently for each biological replicate, and RNA samples that met the quality criteria were used in cDNA synthesis and RT-qPCR analyses. Purified RNA samples were stored at −80 °C until analysis.

#### 2.4.2. RNA Normalization and cDNA Synthesis

To minimize variability that might arise from concentration differences between RNA samples, all RNA samples were normalized to the same concentration using RNase-free water. In total, 1 µg of total RNA was used in each reaction. cDNA synthesis was performed using the OneScript^®^ Plus cDNA Synthesis Kit (Applied Biological Materials Inc., Richmond, BC, Canada) according to the manufacturer’s recommendations. The reverse transcription reaction was performed at 55 °C for 15 min. The reaction was then terminated by incubating at 85 °C for 5 min, and the tubes were immediately placed on ice to stop enzyme activity. The resulting cDNA samples were stored at −20 °C until used in RT-qPCR analyses.

#### 2.4.3. Gene Expression Analysis

Gene expression analyses were performed using the StepOnePlus™ Real-Time PCR System (Applied Biosystems, Foster City, CA, USA). The reaction mixture was prepared according to the manufacturer’s recommendations using Applied Biosystems™ SYBR™ Green Universal Master Mix, and the total volume of each reaction was adjusted to 20 µL. The final concentration for each primer was set to 0.2 µM. Amplification was performed at an annealing temperature of 60 °C. No-template control (NTC) reactions were included in each study to control for contamination. The specificity of the amplification was verified by examining the melting curves obtained at the end of each reaction. Three independent biological replicates were prepared for each of the six isolates, and each biological replicate was analyzed in two technical replicates. Biological replicates were run independently on three different days to allow for the assessment of experimental variability. Relative gene expression was calculated using the 2^−ΔΔCt^ method described by Livak and Schmittgen [27]. Ct values were normalized against the 16S rRNA reference gene (ΔCt = Ct target − Ct reference), and ΔΔCt values were calculated relative to the corresponding 37 °C calibrator condition. Fold-change values were expressed as 2^−ΔΔCt^.

### 2.5. Statistical Analysis

In gene expression analyses, 37 °C was used as the reference (calibrator) condition, representing the optimum growth temperature. The effects of low-temperature stress were evaluated by comparing gene expression at 4 °C and −20 °C with the corresponding 37 °C reference. For heatmap visualization, relative expression values were converted to log_2_ fold-change (log_2_FC), where log_2_FC = −ΔΔCt. Each isolate was independently cultured and sampled on three separate days, representing three biological replicates, and each sample was analyzed in duplicate by RT-qPCR. Technical replicate Ct values were first averaged within each biological replicate. The resulting biological-replicate ΔCt values were then averaged to obtain a single isolate-level ΔCt value for each temperature condition (Supplementary Table S2). Accordingly, the isolate, rather than the individual biological or technical replicate, was treated as the independent experimental unit. Statistical analyses were performed using ΔCt values. For each target gene, the effects of antimicrobial resistance phenotype (susceptible and multidrug-resistant) and temperature condition (37 °C, 4 °C, and −20 °C) were evaluated using two-way repeated-measures ANOVA, with temperature treated as the within-isolate repeated-measures factor. Tukey’s multiple-comparisons test was used for comparisons among temperature conditions within each phenotype, whereas Šídák’s multiple-comparisons test was used to compare susceptible and multidrug-resistant isolates at each temperature. Analyses were performed using GraphPad Prism version 10 (GraphPad Software, San Diego, CA, USA). Results are presented as mean ± standard error of the mean (SEM), and *p* < 0.05 was considered statistically significant.

## 3. Results

### Expression Changes Under Cold and Freezing Stress

**Figure 1:**
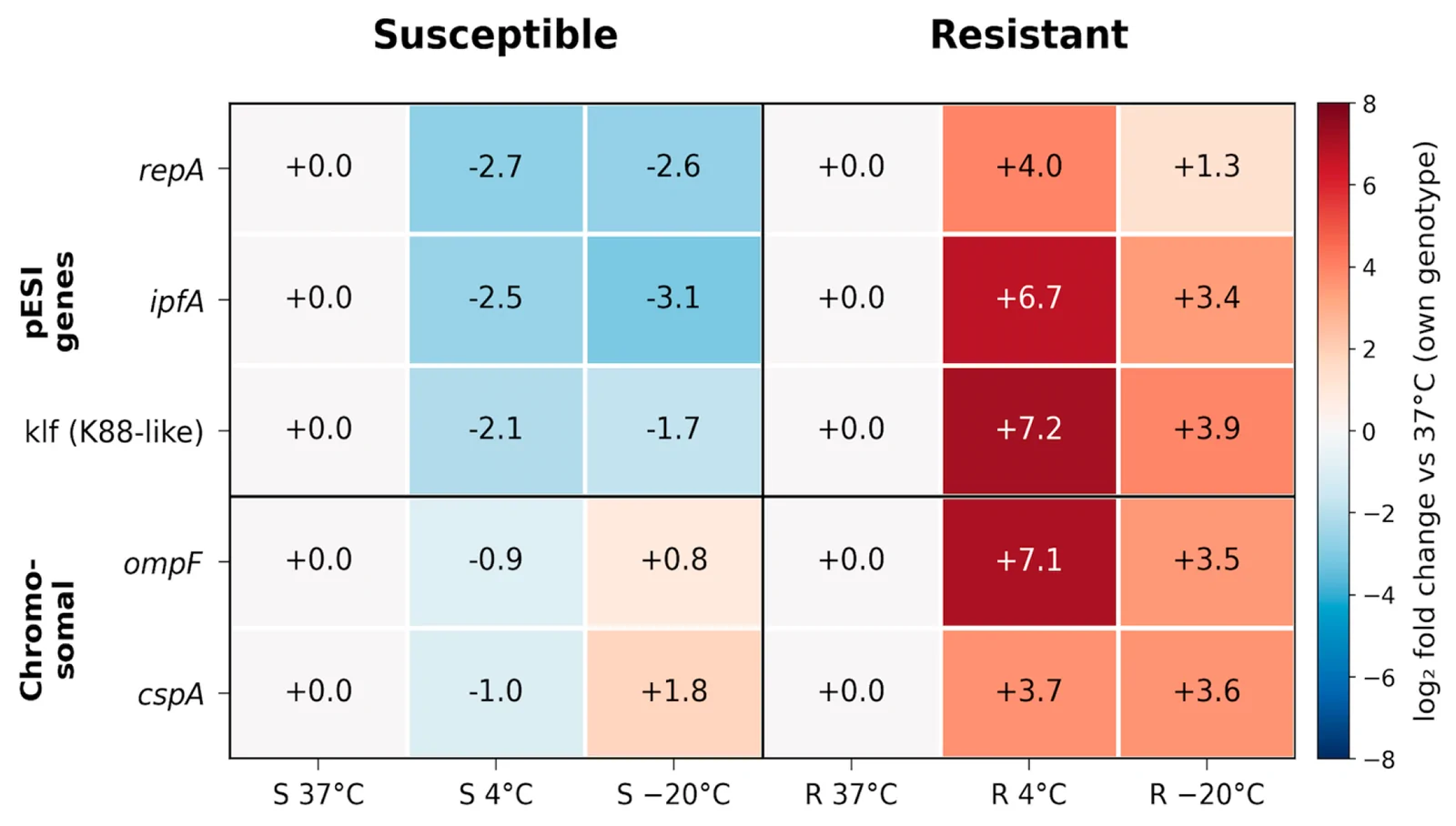
pESI plasmid and chromosomal gene expression under cold and freezing stress. Relative expression of three pESI-encoded genes (*repA*, *ipfA*, *klf*) and two chromosomal stress-response genes (*ompF*, *cspA*) in susceptible and resistant *S.* Infantis isolates. The heatmap shows mean log_2_ fold-change (log_2_FC) values at 4 °C and −20 °C within each isolate group, calculated relative to the corresponding 37 °C calibrator for each isolate. Red: increased expression; blue: decreased expression, relative to 37 °C.

At the group-mean level, opposite directional patterns were observed at 4 °C: mean expression decreased across all five targets in the susceptible group but increased across all five targets in the MDR group relative to their respective 37 °C controls. At −20 °C, group-mean expression was increased across all five targets in the MDR group, whereas the susceptible group showed decreased mean expression of the three pESI-associated targets (*repA*, *ipfA*, and *klf*) but modest increases in the chromosomal genes *ompF* and *cspA*. Thus, the clearest contrast between the group-mean transcriptional patterns was observed at 4 °C (Figure 1). Inspection of the individual-isolate values revealed substantial within-group heterogeneity. R2 did not show the pronounced increases at 4 °C observed for R1 and R3, while S2 showed increased *ompF* and *cspA* expression at 4 °C despite the negative mean values of the susceptible group. Therefore, the group-mean patterns should not be interpreted as uniform responses across all isolates.

**Figure 2:**
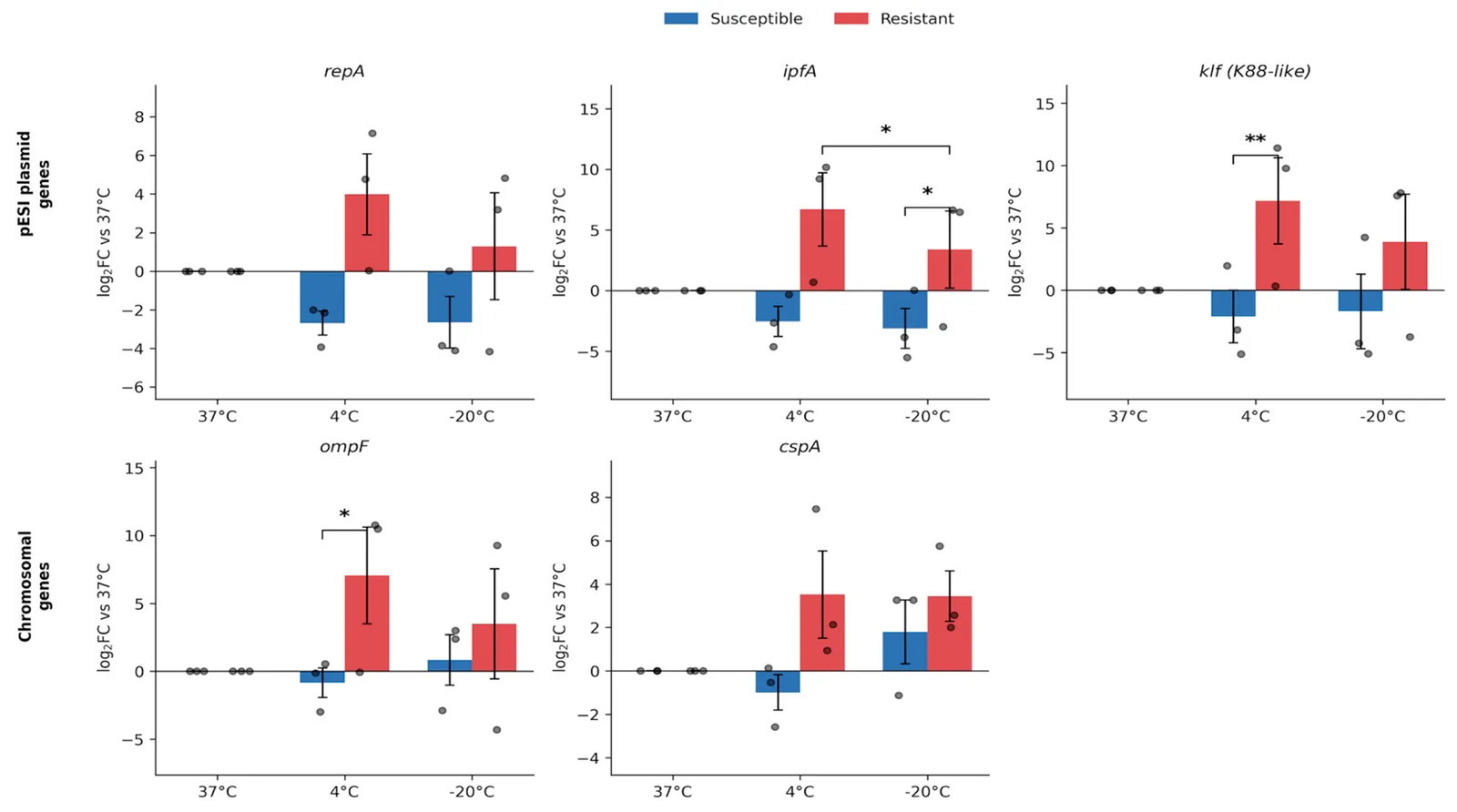
Gene expression changes in the selected isolate groups across temperature conditions. Bars show mean log_2_FC (±SEM) relative to the corresponding 37 °C control within each phenotype. Individual isolate values are overlaid as points. Two-way repeated-measures ANOVA followed by Tukey’s (within-group) or Šídák’s (between-group) multiple comparisons tests. Brackets indicate significant comparisons (* *p* < 0.05, ** *p* < 0.01).

A significant temperature-by-phenotype interaction was detected for *ipfA* (*p* = 0.029), whereas the interactions for *repA*, the K88-like fimbrial locus (*klf*), *cspA*, and *ompF* did not reach statistical significance (*p* = 0.066–0.125). Within resistant isolates, expression at 4 °C differed significantly from −20 °C for *ipfA* (Tukey, *p* = 0.013). Direct comparisons between the susceptible and MDR groups at each temperature identified significant differences at 4 °C for *klf* (Šídák-adjusted *p* = 0.0099) and *ompF* (*p* = 0.0148), and at −20 °C for *ipfA* (*p* = 0.0353). No significant between-group differences were observed for *repA* or *cspA* at either temperature (Figure 2).

Across the ten between-group comparisons, three reached statistical significance: two at 4 °C (*klf* and *ompF*) and one at −20 °C (*ipfA*) (Table 3).

## 4. Discussion

One of the most striking findings of the study was the contrast in the group-mean transcriptional patterns between the selected pESI-positive resistant and susceptible *S.* Infantis isolate groups at 4 °C. While the mean expression of both pESI-associated (*repA*, *ipfA*, and *klf*) and chromosomal (*ompF* and *cspA*) targets increased in the MDR group, mean expression decreased across all five targets in the susceptible group. Parallel to this difference in group-mean patterns, *klf* and *ompF* expression differed significantly between the susceptible and MDR groups at 4 °C. Furthermore, the significant temperature by phenotype interaction for *ipfA* indicated that its expression pattern across temperatures differed between the two selected isolate groups. Although the group means showed opposite directional changes across all five targets at 4 °C, these trends were not uniform across individual isolates.

The similar direction of the group-mean expression changes observed for the pESI-associated *repA*, *ipfA*, and *klf* targets in the MDR group is noteworthy, given that these genes have different functions and regulatory structures. The ipf operon is repressed by Fur and activated by *OmpR*, while the *klf* cluster is negatively and positively regulated by the pESI-derived *KlfL* and *KlfB*, respectively, and activated by chromosomal Lrp [28]. In contrast, *repA* is primarily associated with plasmid replication and maintenance and does not function directly as a transcriptional regulator. Despite these differences, the similar direction of the group-mean changes raises the possibility that factors beyond gene-specific promoter responses, including plasmid copy number or DNA topology, could contribute to the observed patterns. Indeed, Müller et al. [29] reported simultaneous changes between temperature-dependent plasmid copy number alterations and plasmid-derived virulence traits and fimbrial expression in the MDR *Klebsiella pneumoniae* ST307 isolate. However, the fact that the increase in copy number was not reflected in the transcription of all genes on the plasmid indicated that gene dose alone did not determine expression levels. Similarly, it has been shown that transposon-related genes are induced in a coordinated manner in MDR *Acinetobacter baumannii* isolates exposed to antibiotic stress, and this response is associated with the resistance phenotype [30]. Since plasmid copy number and DNA superhelix structure were not directly measured in this study, these mechanisms should be considered as hypotheses to be tested rather than a confirmed explanation.

Aviv et al. [28] reported increased expression of *ipfA* and several *klf* genes at 41 °C relative to 37 °C in a single *S.* Infantis strain grown to stationary phase under microaerobic conditions. In the present study, the group-mean expression of *ipfA* and *klf* increased under low-temperature conditions in the MDR group but decreased in the susceptible group; however, differences in isolate background, temperature range, oxygen conditions, and experimental design preclude a direct mechanistic comparison.

The increased mean expression of *ompF* at 4 °C was one of the notable findings in the MDR group. Decreased expression or loss of function of major porins in Gram-negative bacteria is a well-defined resistance mechanism that can restrict outer membrane permeability, thereby reducing the entry of β-lactams and some fluoroquinolones into the cell [15]. Contrary to this general expectation, *ompF* was not suppressed in resistant isolates but showed increased mean expression at 4 °C; importantly, the difference between susceptible and resistant isolates at this temperature was statistically significant (Šídák-adjusted *p* = 0.0148). However, the increase in *ompF* may not be directly limited to antibiotic permeability but may also be linked to outer membrane stress and osmoregulation requirements caused by cold conditions. The increase in *ompF* expression may instead reflect temperature-dependent outer-membrane adaptation. Indeed, it has been reported that there is no unidirectional association between the expression of *marA*, *soxS*, *ramA*, and *acrB* genes associated with the AcrAB–TolC system and *gyrA* mutations or ciprofloxacin susceptibility in *Salmonella* isolates, and that expression responses vary significantly among isolates [31]. Similar heterogeneity has been observed in the antibiotic susceptibility profiles of *S*. Infantis, *S*. Kentucky, *S*. Schwarzengrund, and *S.* Typhimurium isolates adapted to sanitizer stress after 4 °C cold shock [32]. Therefore, the observed increase in mean *ompF* expression may reflect an isolate-specific response to low-temperature stress, although its underlying mechanism cannot be determined from the present data.

*cspA* showed the weakest between-group differentiation among the five targets examined, with no significant difference between the susceptible and MDR groups at either temperature. In the susceptible group, mean *cspA* expression decreased at 4 °C but increased relative to the 37 °C control at −20 °C. The csp protein family is a key component in the cold adaptation of *Salmonella*. However, this response is time-dependent and may regress to a lower level following rapid induction during the acclimatization period after a temperature drop [12,33]. Therefore, the 4 °C profile measured after 24 h may reflect that the acute *cspA* response is largely complete. The relative transcript increase at −20 °C should be evaluated more cautiously. Because metabolic and transcriptional activity is largely restricted after cell suspension freezing, the amount of *cspA* detected after 24 h may not reflect active transcription continuing during this period. Instead, the signal may reflect the preservation of transcripts formed at the onset of cooling and freezing at low temperatures, or their slower degradation compared to other transcripts. Since early sampling points and measurements of transcript stability are not available, this profile at −20 °C does not allow for differentiation between new transcription and selective mRNA preservation.

This transcriptional pattern also has potential implications for food safety. The proliferation of multidrug-resistant, pESI-positive *S.* Infantis clones, particularly in the poultry food chain, raises concerns about the persistence of these strains. The persistent and multidrug-resistant REPJFX01 clone identified in the United States is a current example of this [34]. A similar picture has been revealed in a genomic surveillance study conducted in the Netherlands. Analysis of 69 *S.* Infantis isolates obtained from retail poultry products between 2008 and 2020 identified six phylogenetic clusters that continued to be detected in different months; the pESI megaplasmid was detected in 77% (53/69) of the isolates and in five of the persistent clusters. The more frequent presence of the plasmid in more recent isolates also supports the increasing importance of pESI-carrying strains in the food chain [35]. On the other hand, cold storage conditions do not guarantee the complete elimination of *Salmonella*. It has been reported that the total number of culturable cells is largely preserved in processed chicken products stored at −20 °C for up to 16 weeks, whereas low counts in selective media indicate sublethal damage developing in the cells [36]. Similarly, it has been shown that *S*. Infantis can be detected for longer periods in cold-stored eggs compared to samples at room temperature [37]. Therefore, although refrigeration and freezing limit bacterial proliferation, it is understood that surviving cells can maintain their presence in the food chain. The group-mean transcriptional pattern observed at 4 °C in the MDR group therefore raises a testable hypothesis regarding its possible relationship with persistence under cold-chain conditions. However, since cell viability or CFU levels were not measured in this study, the observed expression pattern cannot be directly correlated with cold survival capacity. Moreover, as the study focused on a deliberately selected set of three susceptible and three resistant pESI-positive *S.* Infantis isolates, the broader relevance of these transcriptional patterns should be further evaluated in larger and more diverse isolate collections. Future studies combining time-dependent gene expression, CFU and viability measurements, and genomic characterization in larger isolate collections will be required to determine whether these transcriptional patterns are associated with persistence in the cold chain.

## 5. Conclusion

This exploratory study identified different group-mean transcriptional responses to low-temperature stress in the selected pESI-positive *S.* Infantis isolate groups. The clearest difference between the susceptible and MDR group means was observed at 4 °C, although individual-isolate responses were heterogeneous. Resistant isolates showed coordinated increases across plasmid-associated and chromosomal targets, whereas susceptible isolates showed decreases across all five genes. These findings indicate that pESI marker positivity does not correspond to a uniform low-temperature transcriptional response across the selected isolates; however, the relative contributions of antimicrobial resistance phenotype, pESI variation, and genetic background cannot be distinguished from the present dataset. Future studies should combine larger, genomically characterized isolate collections and pESI-negative controls with time-resolved expression, CFU/viability, and plasmid copy-number analyses; small-RNA and epigenetic regulation may also warrant investigation.

## Figures and Tables

**Table 1:** Characteristics of the selected *Salmonella* Infantis isolates.

Isolate	Sample Type	Year	Geographic Origin	Phenotype	*repA*	*ipfA*	K88-like
S1	Chicken liver	2024	Eastern Anatolia, Türkiye	Susceptible	+	+	+
S2	Chicken thigh	2024	Eastern Anatolia, Türkiye	Susceptible	+	+	+
S3	Chicken wing	2024	Eastern Anatolia, Türkiye	Susceptible	+	+	+
R1	Chicken thigh	2024	Eastern Anatolia, Türkiye	Resistant	+	+	+
R2	Chicken thigh	2024	Eastern Anatolia, Türkiye	Resistant	+	+	+
R3	Chicken liver	2024	Eastern Anatolia, Türkiye	Resistant	+	+	+

**Table 2:** Primer Pairs Used in the Study.

Target Gene	Primer	Reference
*repA* F	AAGGCGATGGAGCAACTCAG	
*repA* R	TGCTCCGGTTCCTTTTCCAC	
*ipfA* F	ACTGGTATGCTGTCCCTTGC	[20]
*ipfA* R	TGCTGCAGTCTTGGCAGTAG	
*K88*-like (*klf*) F	TGTATTCCACCCGGATTACTGC	
*K88*-like (*klf*) R	GGCATTTCTCCCGGAATGAGG	
16S rRNA F	CGTGTTGTGAAATGTTGGGTTAA	[24]
16S rRNA R	CCGCTGGCAACAAAGGATAA	
*ompF* F	GTTGAATCCTATACCGATATGG	[25]
*ompF* R	GAGTTAATGCTGTGGTTGTCTTC	
*cspA* F	GGCTTTATTACTCCTG	[26]
*cspA* R	CTTTCTGACCTTCGTCCA	

**Table 3:** Quantitative summary of temperature-dependent expression and phenotype comparisons.

Gene	Temperature	Susceptible log_2_FC ± SEM	Resistant log_2_FC ± SEM	*p* (S vs. R)
*repA*	4 °C	−2.7 ± 0.6	4.0 ± 2.1	0.1268
*repA*	−20 °C	−2.6 ± 1.3	1.3 ± 2.8	0.5365
*ipfA*	4 °C	−2.5 ± 1.2	6.7 ± 3.0	0.0500
*ipfA*	−20 °C	−3.1 ± 1.6	3.4 ± 3.2	**0.0353**
*klf*	4 °C	−2.1 ± 2.1	7.2 ± 3.5	**0.0099**
*klf*	−20 °C	−1.7 ± 3.0	3.9 ± 3.8	0.9993
*ompF*	4 °C	−0.9 ± 1.1	7.1 ± 3.6	**0.0148**
*ompF*	−20 °C	0.8 ± 1.9	3.5 ± 4.1	0.9948
*cspA*	4 °C	−1.0 ± 0.8	3.7 ± 1.9	0.9499
*cspA*	−20 °C	1.8 ± 1.5	3.6 ± 1.1	0.9510

Mean log_2_FC (±SEM relative to 37 °C) for each gene and temperature in susceptible and resistant isolates, with the Šídák-adjusted *p*-value for the direct susceptible-vs.-resistant comparison at that temperature. Values are presented as mean ± SEM for three independent isolates per phenotype group (n = 3). Bold *p*-values indicate *p* < 0.05.

## Data Availability

The original contributions presented in this study are included in the article and Supplementary Materials. Further inquiries can be directed to the corresponding author.

## Supplementary Materials

The following supporting information can be downloaded at: https://www.mdpi.com/article/10.3390/foods15193512/s1. Table S1: Antimicrobial susceptibility profiles and inhibition-zone diameters of the isolates. Table S2: Isolate-level mean ΔCt values of the target genes under different temperature conditions.
