# Supplementary material for: Low-Temperature Transcriptional Responses in Selected pESI-Positive Salmonella Infantis Isolates with Contrasting Antimicrobial Resistance Phenotypes: An Exploratory Study
Source: Foods. 2026 Oct 1;15(19):3512. doi: 10.3390/foods15193512 (PMC13664516; doi:10.3390/foods15193512)
Supplement: Supplementary file 1 [file foods-15-03512-s001.zip › foods-4561940-supplementary.pdf]

Supplementary Table S1

Antimicrobial susceptibility profiles and inhibition-zone diameters of the isolates.

Isolate	AMC	AM	ATM	FEP	CPD	CXM
S1	22 (S)	19 (S)	30 (S)	28 (S)	26 (S)	25 (S)
S2	26 (S)	21 (S)	35 (S)	33 (S)	25 (S)	24 (S)
S3	23 (S)	19 (S)	34 (S)	28 (S)	24 (S)	25 (S)
R1	7 (R)	12 (R)	10 (R)	15 (R)	6 (R)	7 (R)
R2	7 (R)	7 (R)	13 (R)	14 (R)	8 (R)	7 (R)
R3	6 (R)	7 (R)	13 (R)	12 (R)	7 (R)	11 (R)

S, susceptible; R, resistant. AMC, amoxicillin–clavulanic acid (20/10 μg); AM, ampicillin (10 μg); ATM, aztreonam (30 μg); FEP, cefepime (30 μg); CPD, cefpodoxime (10 μg); CXM, cefuroxime (30 μg).

Zone-diameter cutoffs (mm) were taken from EUCAST v13.1 (2023), Enterobacterales: AMC ($S \geq 19$, $R < 19$), AM ($S \geq 14$, $R < 14$), ATM ($S \geq 26$, $R < 21$), FEP ($S \geq 27$, $R < 24$); and CLSI M100-Ed33 (2023), Enterobacterales Table 2A: CPD ($S \geq 21$, $R \leq 17$), CXM ($S \geq 18$, $R \leq 14$)

Supplementary Table S2

Isolate-level mean ΔCt values of the target genes under different temperature conditions.

Isolate	Phenotype	Temperature	*ompF*	*cspA*	*ipfA*	*repA*	*klf* (K88-like)
S1	Susceptible	37°C	9.7475	20.2900	15.7914	12.6969	14.5481
S1	Susceptible	4°C	12.7275	22.8725	16.1093	14.8473	12.5718
S1	Susceptible	−20°C	12.6375	21.4250	15.7531	12.6860	10.3023
S2	Susceptible	37°C	12.1175	22.2825	9.0136	12.1812	6.5729
S2	Susceptible	4°C	11.5625	22.1500	13.6356	14.1930	11.6807
S2	Susceptible	−20°C	9.1275	19.0150	14.5328	16.0364	11.6652
S3	Susceptible	37°C	12.7575	23.8450	11.4630	10.5819	10.0152
S3	Susceptible	4°C	12.8850	24.3800	14.1308	14.5172	13.1966
S3	Susceptible	−20°C	10.3800	20.5850	15.3056	14.6839	14.2493
R1	Resistant	37°C	19.1500	23.0950	18.5954	16.8825	18.9400
R1	Resistant	4°C	8.3825	20.9525	9.3671	12.1208	9.1700
R1	Resistant	−20°C	9.8850	21.0850	12.1186	12.0488	11.3600
R2	Resistant	37°C	7.6500	24.4025	10.0629	8.9636	8.5519
R2	Resistant	4°C	7.7250	23.0625	9.3721	8.9305	8.2157
R2	Resistant	−20°C	11.9675	21.4275	13.0376	13.1192	12.2891
R3	Resistant	37°C	16.6900	24.8575	20.0936	16.4839	19.9415
R3	Resistant	4°C	6.2075	17.3825	9.9279	9.3373	8.5174

Isolate	Phenotype	Temperature	*ompF*	*cspA*	*ipfA*	*repA*	*klf* (K88-like)
R3	Resistant	−20°C	11.1400	19.1100	13.4427	13.2877	12.1162

Values represent the mean ΔCt for each isolate under each temperature condition, calculated as ΔCt = Ct(target gene) − Ct(16S rRNA).
